# Opioid-Induced Constipation in Oncological Patients: New Strategies of Management

**DOI:** 10.1007/s11864-019-0686-6

**Published:** 2019-12-19

**Authors:** Ricard Mesía, Juan Antonio Virizuela Echaburu, Jose Gómez, Tamara Sauri, Gloria Serrano, Eduardo Pujol

**Affiliations:** 1 Servicio de Oncología Médica, Instituto Catalán de Oncología, Badalona, Spain; 2 Servicio de Oncología Médica, Hospital General Universitario Virgen Macarena, Seville, Spain; 3 Hospital la Fe, Valencia, Spain; 4 Hospital Clinic de Barcelona, Barcelona, Spain; 5 Hospital Infanta Leonor, Madrid, Spain; 6 Hospital Clínico Lozano Blesa, Zaragoza, Spain

**Keywords:** Cancer, Opioid-induced constipation, Opioid analgesics, Pain, Naloxegol, Laxatives

## Abstract

Cancer-associated pain has traditionally been treated with opioid analgesics, often in escalating doses. Opioid-induced constipation (OIC) is a common problem associated with chronic use of opioid analgesics. Typical treatment strategies to alleviate constipation are based on dietary changes, exercise, and laxatives. However, laxatives have a nonspecific action and do not target underlying mechanisms of OIC. This article will review prevalent, clinical presentation and recommendations for the treatment of OIC. An independent literature search was carried out by the authors. We reviewed the literature for randomized controlled trials that studied the efficacy of laxatives, naloxone, and naloxegol in treating OIC. Newer strategies addressing the causal pathophysiology of OIC are needed for a more effective assessment and management of OIC. Finally, traditional recommended therapies are appraised and compared with the latest pharmacological developments. Future research should address whether naloxegol is more efficacious by its comparison directly with first-line treatments, including laxatives.

## Introduction

Pain is one of the most common symptoms related with cancer and its treatment [1]. Pain prevalence in patients is 33% after curative treatment, 59% during curative treatment, and 64% in patients with metastases or in advanced stage disease. A multicenter study in Spain has demonstrated that approximately 55% of all cancer patients suffer from pain [2]. Pharmacotherapy, especially opioids, is the principal modality for managing chronic pain in cancer patients. This type of therapy could be related with different adverse effects in these patients. Opioid-induced bowel dysfunction (OIBD) is a frequent complication of long-term opioid treatment, which affects 40–80% of patients treated with opioids. It can cause reduced quality of life (QOL) and insufficient treatment of pain [3]. Opioid-induced constipation (OIC) is the most frequent and bothersome symptom, it affects 60 to 90% of cancer patients with opioids [4], between 10% and 20% of the population experiencing constipation at baseline; however, some of them develop it because of the opioids (OIC), but, in others, constipation is an exacerbation of the pre-existing one (opioid-exacerbated constipation, OEC) [5]. Traditional treatment strategies to alleviate constipation, such as laxatives, are also used to manage OIC; however, laxatives do not address the underlying opioid receptor-mediated cause of OIC and are often ineffective. A novel approach for selectively and locally antagonizing the gastrointestinal effects of opioids involves the coadministration of a peripherally acting μ-opioid receptor antagonist (PAMORA) with negligible availability to the central nervous system (CNS), such as oral naloxegol.

## Materials and methods

An independent literature search was carried out by the authors (during November 2017) with the abovementioned search strategy and the following details were extracted: trial site, year, trial methods, participants, interventions, and outcomes.

We conducted the review protocol using the Preferred Reporting Items for Systematic Reviews and Meta-analyses (PRISMA) guidelines. We used the following search protocol in PubMed: (“Opioid-induced constipation” [All Fields] OR (“Opioid-induced constipation” [Supplementary concept]) OR (“cancer and constipation” [Supplementary concept] OR “cancer and constipation” [All Fields] OR (“Opioid-induced bowel dysfunction” [Supplementary concept] OR “Opioid-induced bowel dysfunction” [All Fields] OR “laxatives” [All Fields]) OR (“laxatives” [Supplementary concept] OR “naloxegol” [Supplementary concept] OR “naloxegol” [All Fields]).

We reviewed the literature for randomized controlled trials that studied the efficacy of laxatives, naloxone, and naloxegol in treating OIC.

The articles were selected following these preferences: English language, human and animal studies, and research support. Case reports have been excluded from this review. The retrieved studies were screened for inclusion and exclusion criteria.

## Results

After applying our research protocol, 83 publications were identified, 20 of which were clinical trials. Also, taken into account were observational studies on laxatives, naloxone, and naloxegol in patients with OIC, in different environments.

The current review starts by providing a brief overview of the pathophysiological mechanisms whereby opioids modify GI function. After addressing the use of laxatives and opioid receptor antagonists for treatments of OIC, the article goes on to discuss emerging strategies to avoid OIBD and the clinical utility of PAMORAs.

### Pain and cancer

Advanced cancer patients frequently suffer from pain at all stages of the disease, during active treatment, during metastatic stage disease, and after the cancer had been cured. Pain is undertreated in 31 to 65% of these patients, although adequate pain relief is considered feasible in 86% of patients with cancer [6]. Although reports vary widely, the range of reported prevalence of pain is highest for the following tumors: head and neck (67–91%); prostate (56–94%); uterine (30–90%); genitourinary (58–90%); breast (40–89%); pancreatic (72–85%); gastrointestinal (44–74%); and lung/bronchus (44–67%) [7].

The cause of pain is a verifiable lesion or disorder that is likely to produce pain through direct tissue injury or through a related process, such as inflammation [8]. Overall, 3 in 4 patients suffer from cancer-related pain while most of the remaining pain syndromes are caused by disease-modifying therapy. Mucosal pain that affects diet occurs during active cancer therapy and is a common chronic complaint in survivors that affects diet and QoL [9].

Opioid analgesics have a significant role to play in the management of chronic pain. They are therefore integrated with strategies that include an interventionist, psychological, physical, or complementary focus to improve the treatment of pain, enabling rehabilitation [10]. Common adverse effects to opioid treatment include nausea, headache, confusion, and gastrointestinal (GI)-related symptoms [11].

### Opioid-induced bowel dysfunction and constipation

GI-related symptoms, normally referred as (OIBD), are the most common and bothersome adverse events of opioid treatment. Up to 80% of patients who receive opiates experience OIBD symptoms such as dry mouth, nausea, acid reflux, loss of appetite, abdominal pain, bloating, and symptoms related to constipation (OIC) [3].

OIBD symptoms are mediated by opioid interaction with peripheral μ-opioid receptors located at the gastrointestinal tract. Enteric opioid receptors (predominantly μ and δ) are located in the submucosal and myenteric plexus, respectively, in addition to immune cells in the lamina propria of the cellular wall. The activation of these receptors inhibits excitatory and inhibitory neural pathways within the enteric nervous system that coordinates motility. These mechanisms reduce peristaltic contractions (due to the inhibition of excitatory neural pathways), increase GI muscle activity, and elevate resting muscle tone, spasm and non-propulsive motility patterns (due to the inhibition of the inhibitory neural pathways). As a result, delayed gastric emptying and intestinal transit slowing is induced [11]. Activation of opioid receptors by opioid administration inhibits secretion of several regulatory neurotransmitters of GI tract, which leads to the discoordination of GI tract motility [12]. Spastic achalasia-like esophageal dysmotility, for instance, is the result of non-peristaltic esophageal contraction with incomplete relaxation of the lower esophageal sphincter after opioid administration. In the small and large intestine, these imbalances lead to increased segmental contraction and decreased propulsive forward peristalsis, which manifest clinically by constipation, gut spasm, and abdominal cramps [13]. In addition, direct activation of μ-opioid receptors (MOP) in the enteric nervous system inhibits vasoactive intestinal peptide (VIP) secretion and subsequently decreases pancreaticobiliary secretion and gut absorption and hence, harder and drier stool.

OIC prevalence rates ranged from 15 to 90% based on an analysis of 16 clinical trials and observational studies identified in patients with or without cancer [14] and from 70 to 100% among hospitalized patients. This variation may be attributable, among others, to patient population assessed, study design, and the lack of standardized definition of constipation across studies. Only few studies have described OIC in cancer patients. 62% of the patientes showed a problematic degree of constipation, according to the Knowles–Eccersley–Scott symptom (KESS) score, in the DYONISOS study (DYsfonctiONs Intestinales induiteS par les OpioıdS forts), a cross-sectional observational study with 520 cancer patients in France [15]. In a study carried out in Spain with 317 ambulatory patients undergoing treatment with different opioids for chronic pain, whether caused by cancer or not, 94.6% of patients reported at least one symptom of OIBD. The most frequent symptom was constipation (91.6%). Almost half the patients reported three or more symptoms with a severity equal to or greater than 4 according to the numeric rating scale (11-point scale, from 0 to 10) [16].

Relevant impairment of QoL in patients with cancer pain and OIC has been reported. Cancer patients with OIC have significantly reduced QoL compared with those cancer patients without OIC, as measured by both the condition-specific Patient Assessment of Constipation–Quality of Life (PAC-QOL) and the generic Short Form-12 questionnaires [15]. OIC negatively affect pain management, productivity, and patients’ QoL [3] and also increase the use of healthcare resources and costs. The risk of having an all cause inpatient hospitalization, emergency department visit, and office or other outpatient visit was nearly twice higher in patients on chronic opioid treatment with OIC [15].

Recently, OIC definition has been proposed as a change, when initiating opioid therapy, from baseline bowel habits and defecation patterns that is characterized by any of the following symptoms: reduced bowel frequency; development or worsening of straining; a sense of incomplete evacuation; or a patient’s perception of distress related to bowel habits [17]. Rome Foundation has endorsed such definition and included OIC as a new bowel disorder, the first OIC diagnostic criteria have been described in the new Rome IV publication. Consensus definition and diagnostic criteria for OIC are now pending adoption to guide clinical and epidemiological research and to inform treatment recommendations.

Patient reported outcome measures are also important to identify OIC. To ensure OIC assessment, patient constipation evaluation scales are recommended. The most commonly used include the Constipation Assessment Scale (CAS), Patient Assessment of Constipation Symptoms (PAC-SYM), Bowel Function Diary, and the Bowel Function Index (BFI) [18]. The American Academy of Pain Medicine along with The American Gastroenterology Association recommend using the BFI to ensure rapid and reliable assessment of OIC in clinical practice [19]. BFI is a practical and clinically responsive tool that has been validated in OIC. The BFI is responsive to changes in symptoms severity and across a broad range of patients. The BFI score range from 0 to 100 points, with higher scores indicating a more severe condition, and ≥ 30 points indicating constipation. Furthermore, a BFI ≥ 30 is considered a criterion to initiate specific pharmacological treatment such as PAMORA for patients on laxative treatment. Failure to first-line treatment should be determined rapidly to provide adequate relief to the patients with OIC [20].

A working group of experts identified the most common barriers for the diagnosis and treatment of OIC [21]. Lack of awareness among doctors about OIC in patients receiving opioid therapy, patients’ embarrassment at revealing their symptoms to doctors, doctors’ inability to ask the patient about constipation, the absence of universal diagnostic criteria, and the need of specific treatment for OIC that alleviate constipation while maintaining central analgesia are some of the main obstacles. These barriers alert us that underdiagnosis and undertreatment of OIC might be a frequent clinical issue among patients with OIC. Temporality is important for the differential diagnosis of OIC, therefore, inquiring patients regarding their bowel habits at the time of initiating opioid therapy, and during the treatment, would help to provide a more successful treatment for their patients with OIC symptoms. Introducing Rome IV diagnostic criteria in clinical practice is also essential.

### Treatments of opioid-induced constipation

Although OIC is one of the most common causes of constipation in cancer patients, there are many other possible factors that can affect bowel movements and exacerbate constipation: cancer itself, cancer treatment, inactivity, poor fluid intake and nutrition, etc. Chemotherapy alters the intestinal microbiome resulting in reduced nucleotide and energy metabolism, cofactors and vitamins, signal transduction, and xenobiotic degradation as well as increased glycan metabolism [22]. This dysbiosis may be one of the mechanisms for chemotherapy mucositis, weight loss, and constipation. When making treatment decisions for OIC, all this potential etiologies and combinations of causes should be taken into consideration.

Anticipating and preventing OIC or treating it when it is mild is always easier. Once constipation is established, management can be much more difficult.

First-line treatments for OIC typically involve laxatives, increased dietary fiber, fluid intake, and exercise; however, these are associated with limited efficacy and do not address the underlying mechanism of OIC [3]. Therefore, implementation of new strategies should focus on more specific and targeted OIC treatment to provide a more satisfactory OIC management in patients with cancer. Furthermore, concomitant therapeutic strategies using traditional therapies and a more specific OIC treatment might be necessary when functional constipation and OIC overlapped.

### Prevention and treatment of constipation

#### Dietary fiber and exercise

There is little evidence that healthy lifestyle changes and exercise improve OIC symptoms; however, data from functional constipation has traditionally been extrapolated to these patients. In healthy subjects, physical activity reduces colon transit time by stimulating colorectal motility. In patients with chronic idiopathic constipation, moderate physical activity 30–60 min per day improves the consistency of stools [23]. Adequate fluid intake is important to promote normal bowel function. However, patients with OIC would not benefit from additional dietary fiber unless their current intake is deficient. If fiber is excessively increased, it might put these patients at risk for bowel obstruction due to the decreased peristalsis, delayed gastric emptying, and prolonged intestinal transit time that occur with OIC [24]. Additionally, side effects of fiber mainly include the production of gas causing abdominal discomfort and flatulence. Soluble fiber pectins, guar, and ispaghula produce viscous solutions in the gastrointestinal tract that slow absorption in the small intestine. This may reduce the absorption of cholesterol and inhibit the activity of the pancreatic enzyme and the digestion of protein, leading to an anti-nutritive effect.

#### Laxatives

The routine prescription of laxatives for prophylaxis and treatment of intestinal dysfunction induced by opioids in cancer patients is recommended by the European Society of Medical Oncology (ESMO) [25•] and by the European Expert Consensus [26], that indicates that prophylactic treatment of OIC with laxatives can be considered, although more supporting evidence is needed.

Laxatives have shown to be effective to manage functional constipation, but OIC and functional constipation have different physio-pathological mechanism [27]. Laxatives may help defecation trough localized effects in the colon, but do not directly address the spectrum of the underlying mechanisms of OIC. There is limited evidence of laxative efficacy in the treatment of OIC. No randomized, controlled, double-blinded trials investigating the efficacy of conventional laxatives in OIC patients have shown superiority of one laxative over the other [28]. A prospective open-label study has suggested that polyethylene glycol and sodium picosulfate might be better than lactulose for cancer patients with opioids [29]. Various studies have demonstrated that laxatives do not alleviate OIC symptoms in some patients and may be associated with adverse events such as bloating, flatulence, sudden urge to defecate, electrolyte imbalances, dehydration and bowel obstruction, which can affect daily activities [3, 30].

Table 1 shows the benefits and side effects of the laxatives investigated in patients with OIC.

**Table 1:** Effect of laxatives in patients treated with opioids

Reference	Study design	Patient population	Dosing regimen	Efficacy outcome
Emmanuel et al. 2017 [30]	A cross-sectional online or telephone survey	Patients who had taken opioid analgesics (*n* = 198, 100%). Patients with cancer (*n* = 3, 1.5%)	1. Patients used osmotic laxative 2. Patients used stimulant laxative	Laxative side effects were reported in 75%, most commonly gas, bloating/fullness, and a sudden urge to defecate. As assessed by the Bowel Function Index, laxatives did not improve the symptoms of constipation
Hawley and Byeon 2008 [31]	Nonrandomized, nonblinded sequential cohort study	Patients with cancer who had taken opioid analgesics (*n* = 60).	1. Thirty patients received the sennosides-only (S) protocol (17.2–25.8 mg) 2. Thirty patients received the sennosides (17.2–25.8 mg) plus docusate (DS) protocol (200 mg)	In the docusate plus sennosides protocol, 57% cancer patients were requiring additional interventions when compared with cancer patients following the sennosides alone protocol (40%)
Agra et al. 1998 [32]	Randomized, open trial	Patients with terminal cancer (taking codeine or morphine) (*n* = 91)	1. Treated with senna (min 12 mg, max 48 mg), (*n* = 43) 2. Treated with lactulose: (min 10 g, max 40 g), (*n* = 48)	The number of defecation days was similar in both groups (senna: mean, 8.9 days; lactulose: mean, 10.6 days). Findings do not provide evidence of the greater efficacy of senna over lactulose in terminal cancer patients treated with opioids
Christensen et al. 2016 [33]	Cross-sectional online survey	Participants treated with opioid for a minimum of 4 weeks and confirmed ever experiencing OIC (*n* = 417)	Both prescribed and non-prescribed laxative users were asked to specify what type of laxatives they bought. All laxative users were categorized according to number of different brands of laxatives used (one laxative, two or more laxatives), frequency of laxative use (daily 6–3 days/week; 1–2 days/week)	Less than half (48%) of the laxative users were satisfied with the laxative they were using to relieve their constipation
Coyne et al. 2014 [34]	Cross-sectional patient survey and chart review data from the baseline assessment of an ongoing longitudinal study	Participants who had confirmed of daily opioid therapy ≥ 30 mg for ≥ 4 weeks and self-reported opioid-induced constipation (*n* = 617)	Both prescribed and non-prescribed laxative users (osmotic, stimulants, stool softeners)	Of patients who took the recommended dose of laxatives, 94% did not get an adequate response.
Tarumi et al. 2013 [35]	Prospective, randomized, double-blind, placebo-controlled trial	Hospice patients and had a Palliative Performance Scale score of 20% or more (*n* = 74)	1. The docusate group received two 100 mg docusate (dioctyl sodium sulfosuccinate) tablets twice daily plus one to three sennoside tablets (8.6 mg/ tablet) 2. The placebo group received two cornstarch capsules twice daily, in addition to one to three sennoside tablets (8.6 mg/tablet)	There was no significant benefit of docusate plus sennosides compared with placebo plus sennosides in managing constipation in hospice patients

A study by Kumar et al. [36] observed that 54% of patients being treated with opioids and laxatives did not achieve the desired level of symptom (constipation) improvement more than half the time.

A failure of laxative treatment can be determined by a BFI score > 30 points, and OIC treatment with PAMORA can be considered [25•, 26].

#### Drugs for opioid-induced constipation

Several recent treatment guidelines recommend taking into account therapies based on peripherally acting opioid antagonists when starting opioid therapy or in patients with OIC who do not respond to laxatives [25•, 26, 28]. In the process of drug development, the initial strategy was to investigate the use of the μ-opioid receptor antagonist naloxone for the treatment of OIC [37].

#### Peripherally acting μ-opioid receptor antagonists

The objective of PAMORAs treatment is to reestablish intestinal function. PAMORAs bind exclusively to the peripheral μ-opioid receptors avoiding opioid union to them. The therapeutic mechanisms of action imply a selective and competitive binding of the drug to the enteric μ-opioid receptors [38]. PAMORAs are the best option treatment to alleviate OIC symptoms without affecting central analgesia [38]. Available PAMORAs include naltrexone, alvimopan, methylnaltrexone, naloxegol, and naldemedine. All PAMORAs have demonstrated efficacy in the treatment of OIC [36]. Rauck et al. [39], in a randomized, double-blind trial showed that oral formulation of methylnaltrexone is efficacious and well-tolerated for OIC. Naldemedine is a novel PAMORA recently approved for the treatment of OIC [40–45]. Spontaneous BM is achieved earlier with naldemedine (16.1 or 18.3 h) than with placebo (46.7 or 45.9 h, respectively) [41]. Song et al. [43] in a meta-analysis have suggested that naldemedine can improve the number of patients achieving spontaneous BM and its frequency. Furthermore, authors reported a higher incidence of serious adverse events in patients receiving naldemedine than placebo, mainly in cancer patients.

Merchan et al. [46] in a recent study compared the effectiveness and safety of oral naloxone versus subcutaneous methylnaltrexone in a medical intensive care unit. Both agents showed similar results on efficacy and safe. The time to first BM for oral naloxone and subcutaneous methylnaltrexone were 30 and 24 h.

#### Naloxegol

Naloxegol is a PEGylated derivative of the opioid antagonist naloxone [4, 38]. Pegylation converts naloxegol to a substrate for the p-glycoprotein transporter (P-gp); this reduces central passive permeability compared with naloxone. Due to the reduction in permeability and the increase in the flow of naloxegol through the hematoencephalic barrier, related to the P-gp transporter, the penetration of the naloxegol into the central nervous system is negligible. It was shown that cerebral entry with naloxegol was insignificant as it did not modify morphine-induced miosis in 47 of 48 patients being treated with oral naloxegol [47]. Naloxegol antagonizes the μ-receptor in the GI tract, decreasing the OIC effects without reversing the central analgesic effect [38]. Naloxegol have shown to mitigate the gastric peristaltic effects of morphine without significantly affecting analgesia [38]. Naloxegol has shown to be effective not only against placebo but also by indirect comparisons with other interventions, and naloxegol has been neither associated with an increased risk of severe adverse events nor with a reduction in analgesia of background opioid analgesic drug [38]. Most relevant studies of naloxegol are shown in Table 2.

**Table 2:** Summary of the most relevant naloxegol studies. Details of relevant naloxegol studies

Author/year	Webster et al. 2013 [49]	Chey et al. 2014 [50]
Phase	II	III
N	*N* = 207; ≥ 18 years old; oral morphine 30–1000 mg	KODIAC-04 *N* = 652; KODIAC-05 *N* = 700; 18–84 years old; oral morphine 30–1000 mg > 4 weeks with good pain control and no cancer diagnosis, GI obstruction or increased risk for bowel perforation who had confirmed, active OIC
Study design and dosing	Multicenter, randomized, DB, placebo-controlled, dose-escalation; 5 mg, 25 mg, and 50 mg orally in sequential cohorts with placebo-control	Multicenter, randomized, DB, parallel-group, placebo-controlled. Naloxegol 12.5 mg, 25 mg, or placebo for 12 weeks
AE	Abdominal pain, diarrhea, and nausea with increased frequency and severity in 50 mg group; no evidence of opioid withdrawal or worsening pain	Diarrhea, abdominal pain, nausea, vomiting; no evidence of opioid withdrawal or worsening pain. More common in 25 mg group
Variables efficacy and outcomes	Primary endpoint: -Change in spontaneous bowel movements (BMs)/week over baseline at the end of week 1 statistically significantly improved compared with placebo at 25 mg and 50 mg doses **Secondary endpoints:** -Change over baseline across weeks 2, 3, and 4 -Change over baseline at the end of week 4 -Time after first dose of naloxegol to first laxation -Improvement over baseline compared with placebo maintained in 25 mg and 50 mg cohort over 4 weeks.	**Primary endpoint**: -Twelve-week response rate (≥ 3 spontaneous BMs/week and ≥ 1 spontaneous BMs over baseline for ≥ 9/12 weeks and ≥ 3/4 of the final weeks) -Higher response for 25 mg group over placebo for both trials and for 12.5 mg group over placebo in KODIAC-04 trial

The Ki values of naloxegol at the cloned human μ-opioid receptor ranged from 6.5 to 8.5 nM. The pKi values of naloxegol and methylnaltrexone corresponded to respective geometric mean Ki values of 7.42 nM and 22.1 nM, showing that naloxegol bound human μ-opioid receptors with three-fold greater affinity than methylnaltrexone [47]. Floettmann et al. [48••] described the results of several studies that employed standard pharmacologic measures of opioid activity and pharmacokinetic measures of CNS and demonstrated that naloxegol relieves OIC-associated symptoms in patients with chronic noncancer pain.

Naloxegol does not significantly inhibit the activity of CYP1A2, CYP2C9, CYP2D6, CYP3A4, CYP2C19, P-glycoprotein, breast cancer resistance protein, organic anion-transporter (OAT)1, OAT3, organic cation transporter-2, organic anion-transporting polypeptide (OATP)1B1, or OATP1B3, nor does it significantly induce the activity of CYP1A2, CYP2B6, or CYP3A4 [41].

A pooled analysis [51] of patients being treated with laxatives for 4 days showed that those with an inadequate response to one or more types of laxatives had response rates of 42.5% and 47.7% for naloxegol 12.5 and 25 mg/day vs those receiving a placebo (30.1%) (*p* = 0.005 and *p* < =0.001, respectively) and those with an inadequate response to two or more types of laxatives had response rates of 44.3% and 44.4% versus 30.0%, respectively (both *p* = 0.05).

To rule out any adverse cardiac events noted with its predecessor alvimopan, an additional trial was conducted (NCT01325415). A randomized, placebo-controlled crossover thorough the QT/QTC study with therapeutic (25 mg) and supratherapeutic (150 mg) naloxegol or moxifloxacin 400 mg or placebo in health volunteers demonstrated no significant cardiovascular changes [52]. In addition, a new clinical trial (NCT03087708) was started in University of Minnesota, to determine feasibility and safety of long-term administration of two doses of naloxegol in patients with advanced NSCLC receiving first-line pemetrexed-based chemotherapy.

Naloxegol has been approved in the European Union for any patient (cancer patient and noncancer patient) suffering with OIC and with an inadequate response to at least one laxative after at least 4 days treatment in the 2 weeks previous to the diagnosis.

In addition SEOM recommend the use of naloxegol in cancer patients with OIC and an inadequate response to first-line treatments (e.g., dietary changes and over-the-counter laxatives) [53••].

#### Additional agents and mechanisms to OIC control

Linaclotide is a peptide agonist of the guanylate cyclase-C receptor involved in the intracellular conversion of guanosine 5-triphosphate to cyclic guanosine monophosphate [54]. Linaclotide has been shown to be an effective and well-tolerated agent for the treatment of chronic constipation [55–58]. Lubiprostone is a specific activator of the type 2 chloride channels at the intestinal epithelium whose activity increases the liquidity of contents [59]. Compared with placebo, lubiprostone has demonstrated to increase spontaneous BM frequency and response rate, and to improve OIC-related symptoms [60–64]. Prucalopride is a highly selective serotonin receptor agonist showing gastrointestinal prokinetic activity. Prucalopride has demonstrated activity in chronic constipation, and European guidelines actually recommend its use [56].

## Conclusion

Pain is a major problem in all stages of cancer. Analgesic drugs are used to manage chronic pain and as part of a multifaceted focus that integrates strategies with an interventionist, psychological, physical, or complementary approach, seeking to improve the treatment of pain and enabling rehabilitation. A common consequence of opiate use, due to the distribution of μ-receptors, is OIC.

Although the evidence is limited and may present some contraindications, the routine prescription of laxatives is recommended for patients who receive opioid analgesia. In some studies, it can be observed that laxatives do not address OIC symptoms in some patients and can cause adverse events that affect daily activities such as bloating, flatulence, and a sudden urge to defecate. Furthermore, laxatives assist defecation through localized effects in the colon, while OIC arises following stimulation of the enteric μ-opioid receptors. Although traditional treatment such as laxatives or the increase of dietary fiber is often insufficient to relieve OIC symptoms, a series of new and more specific pharmacological approaches have emerged. Several treatment guidelines recommend taking into account strategic therapies based on PAMORAs when starting opioid therapy or in patients with OIC who do not respond to laxatives.

Among currently available PAMORAs, daily administration of oral naloxegol seems to be the most appropriate. Naloxegol is effective for the treatment of OIC who do not respond to traditional laxatives. Naloxegol has shown response rates significantly higher versus placebo in the overall patient population and in patients classified as laxative-inadequate responder treated with 25 mg for both studies and 12.5 mg for one study, in two randomized, placebo-controlled, phase 3 trials.

Naloxegol present a well-tolerated safety profile. Adverse events are more frequently GI and mild to moderate in severity and occurred at the time of initiation of naloxegol treatment. Severe adverse events are rare, and no bowel perforation has been reported, differing of what was noticed in seven patients treated with subcutaneously administered methylnaltrexone.

Altogether, naloxegol should be considered as an important alternative for the treatment of OIC in cancer patients. Future research should address whether naloxegol is more efficacious by its comparison directly with first-line treatments, including laxatives.
